# Impacts of predicted climate change on recruitment at the geographical limits of Scots pine

**DOI:** 10.1093/jxb/ert376

**Published:** 2013-11-12

**Authors:** Luis Matías, Alistair S. Jump

**Affiliations:** Biological and Environmental Sciences, School of Natural Sciences, University of Stirling, Stirling FK9 4LA, UK

**Keywords:** Biomass, distribution, emergence, *Pinus sylvestris*, precipitation, range limit, root:shoot, survival, temperature, trade-off.

## Abstract

Ongoing changes in global climate are having a significant impact on the distribution of plant species, with effects particularly evident at range limits. We assessed the capacity of *Pinus sylvestris* L. populations at northernmost and southernmost limits of the distribution to cope with projected changes in climate. We investigated responses including seed germination and early seedling growth and survival, using seeds from northernmost (Kevo, Finland) and southernmost (Granada, Spain) populations. Seeds were grown under current climate conditions in each area and under temperatures increased by 5 °C, with changes in precipitation of +30% or –30% with reference to current values at northern and southern limits, respectively, in a fully factorial controlled-conditions experimental design. Increased temperatures reduced germination time and enhanced biomass gain at both range edges but reduced survival at the southern range edge. Higher precipitation also increased survival and biomass but only under a southern climate. Seeds from the southern origin emerged faster, produced bigger seedlings, allocated higher biomass to roots, and survived better than northern ones. These results indicate that recruitment will be reduced at the southernmost range of the species, whereas it will be enhanced at the northern limit, and that the southern seed sources are better adapted to survive under drier conditions. However, future climate will impose a trade-off between seedling growth and survival probabilities. At the southern range edge, higher growth may render individuals more susceptible to mortality where greater aboveground biomass results in greater water loss through evapotranspiration.

## Abstract (precis)

Scots pine recruitment is likely to change in response to climate variations. A recruitment reduction is expected at the southern edge and an expansion through northern range. However, local adaptations to drought of southern populations might modulate this declining trend

## Introduction

Plant distributions are strongly determined by climate (Houle, 1994; Walck *et al.*, 2011). Temperature and precipitation are critical drivers for plant distribution at the global scale (Woodward, 1987) because they strongly influence seed germination, growth, and survival at the population scale (Arft *et al.*, 1999; Diemer, 2002; Walck *et al.*, 2011). Thus, global alterations in climate as expected for the coming decades (IPCC, 2007) will undoubtedly affect population dynamics throughout the world (Sykes and Prentice, 1995; Thuiller *et al.*, 2008). The geographical limits of species distributions are generally considered more susceptible to changes in climate as ecological conditions there are often already at the limit of the species’ tolerance (Hampe and Petit, 2005). This ecological marginality can result in lower relative fecundity and lower local population densities (Case and Taper, 2000), which could result in reduced resilience under adverse climate conditions. However, population dynamics at range edges are driven by different factors. Poleward expansion of temperate plant species is frequently limited by low temperatures, whereas the equatorial range edge is limited by a combination of drought and high temperatures (Hampe and Petit, 2005). Thus, the expected global temperature rise and alteration of precipitation patterns will have contrasting results at both limits, resulting in range expansions and/or contractions for many species (Matías and Jump, 2012).

There are three possible fates for populations in a rapidly changing environment: persistence through migration to track ecological niches spatially, persistence through adaptation to new conditions in current locations, and local extinction (Aitken *et al.*, 2008). For forest trees, latitudinal and elevational shifts in species ranges have already been recorded in response to climate change (Root *et al.*, 2003; Dobbertin *et al.*, 2005; Hickling *et al.*, 2006; Pauli *et al.*, 2007).However, although species ranges are often viewed as relatively homogeneous, the consequences of changing environmental conditions will differ throughout a species’ distribution due to phenotypic plasticity or local adaptations to specific environmental conditions (Howe *et al.*, 2003; Jump and Peñuelas, 2005; Savolainen *et al.*, 2007, 2011). Numerous experiments have revealed high inter-population levels of genetic variation for quantitative traits related to adaptation, geographical structuring of that variation along climatic gradients, and genotype×environment interaction, providing strong evidence of local adaptation of populations to climate (reviewed by Savolainen *et al.*, 2007; Aitken *et al.*, 2008; Reich and Oleksyn, 2008). Given that the current rate of climate change will challenge the adaptive capacity of many long-lived species as trees, probably exceeding their migration capacity (Rehfeldt *et al.*, 2002; Jump *et al.*, 2009*a*), existing local adaptations that allow individuals to tolerate environmental conditions are especially important in order to increase the resistance and resilience of current populations. However, despite the importance of this topic, there are few experimental studies assessing both the speed and direction of change in colder and warmer regions of a species range as a response to future climate alterations (Reich and Oleksyn, 2008).

Recruitment is often considered the controlling factor driving population dynamics, especially in temperate areas (Houle, 1994; Castro *et al.*, 2005; Rickebusch *et al.*, 2007). Seedlings and young saplings are more sensitive to abiotic conditions than trees because of their limited root system (Houle, 1994), so they respond more rapidly to environmental changes than adult trees (Lloret *et al.*, 2009). For this reason, early plant stages are expected to represent a major bottleneck in plant regeneration over the coming decades (Lloret *et al.*, 2004; Matías *et al.*, 2011*a*). Consequently, information on regeneration under climate change is urgently needed for modelling and predicting vegetation dynamics (Leishman *et al.*, 1992; Ibáñez *et al.*, 2007; Morin and Thuiller, 2009). Species with a wide distribution are especially useful to test changes in recruitment patterns as a consequence of climate alterations, as they grow under strongly different ecological conditions across their range. Scots pine (*Pinus sylvestris* L.) has the largest geographical distribution among pine species, and is one of the most widespread conifers on earth, distributed from the Mediterranean to the Arctic (Carlisle and Brown, 1968). This wide latitudinal distribution results in this species growing under strongly differentiated environmental conditions throughout its range, with the greatest contrast at northern and southern range edges (Matías and Jump, 2012). Consequently, Scots pine is an important study species for the detection of variation in recruitment patterns across wide geographical areas.

The aim of this study was to assess the capacity of the early life stages of the populations from the distribution limits of Scots pine to tolerate and persist under the increased temperatures and altered water availability as projected by climate change scenarios across its distributional range. We focused primarily on seed germination and early seedling growth and survival, as they are the most vulnerable life history stages of trees. We hypothesized that, at its southernmost distribution limit, predicted changes in climate would result in a recruitment bottleneck, whereas recruitment would increase at the northernmost limit. At the same time, we assessed whether local adaptations of the northern and southern populations might enhance population persistence. Use of controlled environment growth chambers allowed precise manipulation of soil moisture and temperature, whilst keeping all other climatic factors such as radiation intensity, daily sunshine duration, and air humidity at constant values. Specifically, we posed the following questions: (i) Do populations of Scots pine at northern and southern range limits show strongly divergent responses to future conditions? (ii) Do the forecast changes in temperature and precipitation affect seedling growth and survival at range limits in a similar way? (iii) Are there local adaptations of the populations from the range limits to climate conditions that might mitigate the possible negative effect of a changing climate?

## Materials and methods

### Seed source

Scots pine has a wide latitudinal distribution through the northern hemisphere ranging from 37°N to 70°N. To determine the consequences of climate variations at the range limits, we selected different populations at both latitudinal limits of the species. Mature cones were collected from three different populations at both northern and southern range limits (Table 1), selecting at least 15 mature trees per population and 10 cones per tree. Seeds were extracted by oven drying the cones at 45 °C for 48h, and then stored at 4 °C until sowing.

**Table 1.** Main characteristics of the selected populations for seed collection at the northern and southern range limits of the study speciesAltitude values are given in metres above sea level (m a.s.l.). Mean values of seed mass (in g) for the different populations are given ±SD (*n*=100). Different letters denote significant differences among populations.

Range	Site	Population	Location	Altitude	Collection year	Seed mass (g)
Northern	Kevo, Finland	1	69°48’06’’N–27°04’40’’E	217	2010	0.0071±0.001 ab
	Kevo, Finland	2	69°47’11’’N–27°03’53’’E	230	2010	0.0067±0.001 a
	Kevo, Finland	3	69°46’41’’N–26°58’17’’E	189	2010	0.0075±0.002 b
Southern	Sierra Nevada, Spain	1	37°05’32’’N–3°27’28’’W	1825	2011	0.0110±0.002 c
	Sierra de Baza, Spain	2	37°22’48’’N–2°51’37’’W	2010	2011	0.0106±0.002 c
	Sierra de Baza, Spain	3	37°21’59’’N–2°52’12’’W	2100	2011	0.0107±0.002 c

### Experimental design

The experiment was conducted using Snijders Scientific (Tilburg, Netherlands) MC1750E controlled environment chambers at the University of Stirling (UK). Four different chambers (1.8 m long×0.75 m wide×1.2 m high) were used to simulate the four different temperature scenarios. In order to test the possible effect of climate alterations on the recruitment pattern at the species’ range limits, we designed an experiment with three main factors: (i) seed provenance: with two levels (Table 1), northern and southern; (ii) temperature: with four different levels (Table 2), current main temperature during growing season at the northern limit of the species (north current, NC), predicted temperature by 5 °C at the northern limit for the end of the present century (north future, NF), current main temperature during growing season at the southern limit of the species (south current, SC), and predicted temperature by 5 °C at the southern limit for the end of the present century (south future, SF); and (iii) precipitation: mean precipitation at northern populations during growing season (May–September, 115mm), expected increase in precipitation at the northern limit by 30% (149.5mm), mean precipitation at southern populations during growing season (220mm), and expected reduction in precipitation at the southern limit by 30% (155mm). As the two levels resulting from the increase and reduction of precipitation at northern and southern limits were highly similar (149.5 and 155mm), they were combined, giving three experimental levels: high (220mm), medium (150mm), and low (115mm). These total precipitation amounts were applied throughout the duration of the experiment by watering twice weekly. Current temperature and precipitation values are based on mean values of field data from the population origin during the growing season (1990–2010 period), whereas future values are based on the projected changes for the 2090–2099 period in those areas (A2 scenario; IPCC, 2007). Light intensity was fixed at a photosynthetic photon flux density of 210 µmol m^–2^ s^–1^ for 16h, which is a representative value for forest understory (Valladares *et al.*, 2004), rising progressively at dawn and decreasing at dusk for 1h, and relative humidity was kept constant at 65%.

**Table 2.** Temperatures (day/night) during the experiment development in the different scenarios: northern current (NC), northern future (NF), southern current (SC), and southern future (SF)

Week	Equivalent	Temperature scenarios (°C)			
		NC	NF	SC	SF
1–2	15–31 May	4.0/0.5	9.0/5.5	12.5/6.5	17.5/11.5
3–6	June	10.5/2.0	15.5/7.0	18.0/10.0	23.0/15.0
7–10	July	12.5/4.5	17.5/9.5	21.5/14.1	26.5/19.1
11–20	August	12.5/4.5	17.5/9.5	21.5/14.2	26.5/19.2

All main factors were crossed in a fully factorial experimental design, with nine replicates for each combination, giving a total amount of 216 plots. Each plot was formed by a 15×15×25cm pot filled with a peat and river sand mixture (proportion 2:1 by volume, respectively) with a layer of gravel at the bottom to facilitate drainage. To allow mycorrhization, all plots were irrigated prior to sowing with 300ml of a soil microbial inoculum obtained from two different fractions: 150ml of a filtered solution resulting from the maceration of 3.5kg of fresh soil collected under Scots pine trees at the University of Stirling campus in 35 l of water over 2 d, and 150ml of a solution derived from the maceration of 1kg of Scots pine fine roots in 35 l of water.

Seeds from the different populations within provenance were combined, and 10 seeds per provenance were then selected at random and sown per plot on 13 January 2012. Each temperature level was assigned to one of the four chambers (blocks), randomly assigning the provenance and irrigation levels within this block. To avoid any possible chamber effect, all pots were rotated through the different chambers, spending at least 1 month in each, whilst also randomizing pot position within chambers. Seedling emergence was recorded daily until after 2 weeks with no new seedlings detected; thereafter, survival was recorded weekly until the end of the experiment. Soil moisture was measured fortnightly during the experiment in all plots over the surface 5cm by a time-domain reflectometry method (SM300; Delta-T devices, Cambridge, UK); values were recorded 2 days after irrigation events. On 28 May 2012, after 20 weeks of the experiment, all surviving seedlings were harvested. Roots were washed carefully to remove soil remains, and maximum root and shoot fresh length were measured. The seedlings were then divided into aboveground and belowground parts, oven dried at 70 °C for 72h and weighed. As the root collar is not always detectable in Scots pine seedlings, we defined ‘aboveground’ as all plant parts growing above the soil surface (Richter *et al.*, 2012).

### Data analysis

Variation in soil moisture among the different treatments was tested by a repeated-measures analysis of variance (ANOVA). We used emergence time (number of days from sowing to emergence), emergence proportion (the proportion of sown seeds from which seedlings emerged), survival proportion (the proportion of emerged seedlings that survived to the end of the experiment), total biomass, and biomass allocation (root:shoot ratio) as response variables. To avoid pseudoreplication, we used the mean values of the different variables from all seedlings growing in the same plot for the different analyses. Differences in emergence time and proportion across treatments were tested by ANOVA and a generalized linear model with binomial error distributions, respectively (Quinn and Keough, 2002). Seedling survival was analysed using a failure-time approach, which measures the time to death of each individual seedling (Fox, 2001), defined as the number of days from emergence until death. When seedling death did not occur before the end of the experiment, we considered its survival time to be the last day of the experiment and we labelled the individual as right censored. We tested the effects of temperature, precipitation, and provenance on survival, using a log-rank test to estimate differences among curves (Fox, 2001). Finally, total biomass and root:shoot ratio were tested by ANOVA tests. Spearman’s rank correlation was used to test for specific relationships among biomass and survival. All variables were transformed (log, arcsin) to meet normality assumptions when was necessary. Results are given as means ±standard error (SE) throughout this paper.

## Results

**Fig. 1.**
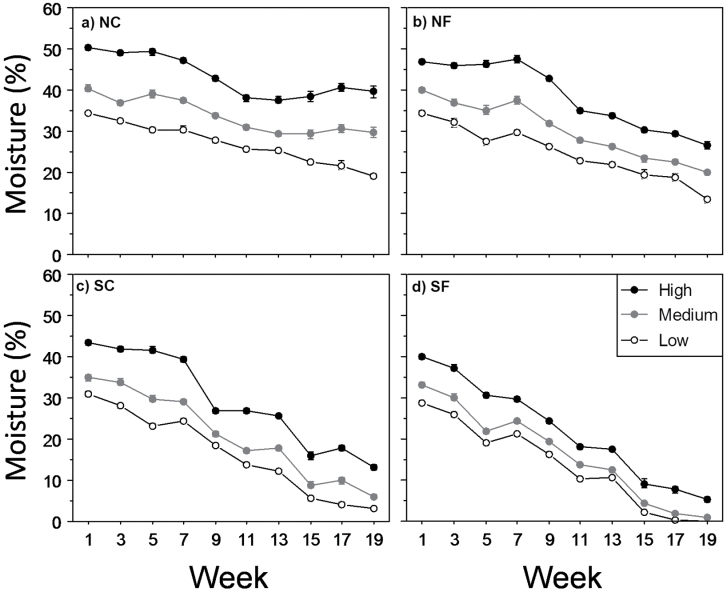
Soil moisture (volumetric water content, %) during experiment development under the different precipitation (high, black circles; medium, grey circles; low, white circles) and temperature levels: NC (a), NF (b), SC (c), and SF(d). Results are given as means ±SE.

Precipitation levels imposed during the experiment resulted in different soil moisture levels across treatments. Water availability was significantly different across temperature (*F*_3,192_=1405.6; *P*<0.0001) and precipitation levels (*F*_2,192_=1185.6; *P*<0.0001), but not between provenances (*F*_1,192_=0.8; *P*=0.36) (Fig. 1). In addition, a significant interaction occurred between temperature and precipitation (*F*_6,192_=21.9; *P*<0.0001).

### Seedling emergence

**Fig. 2.**
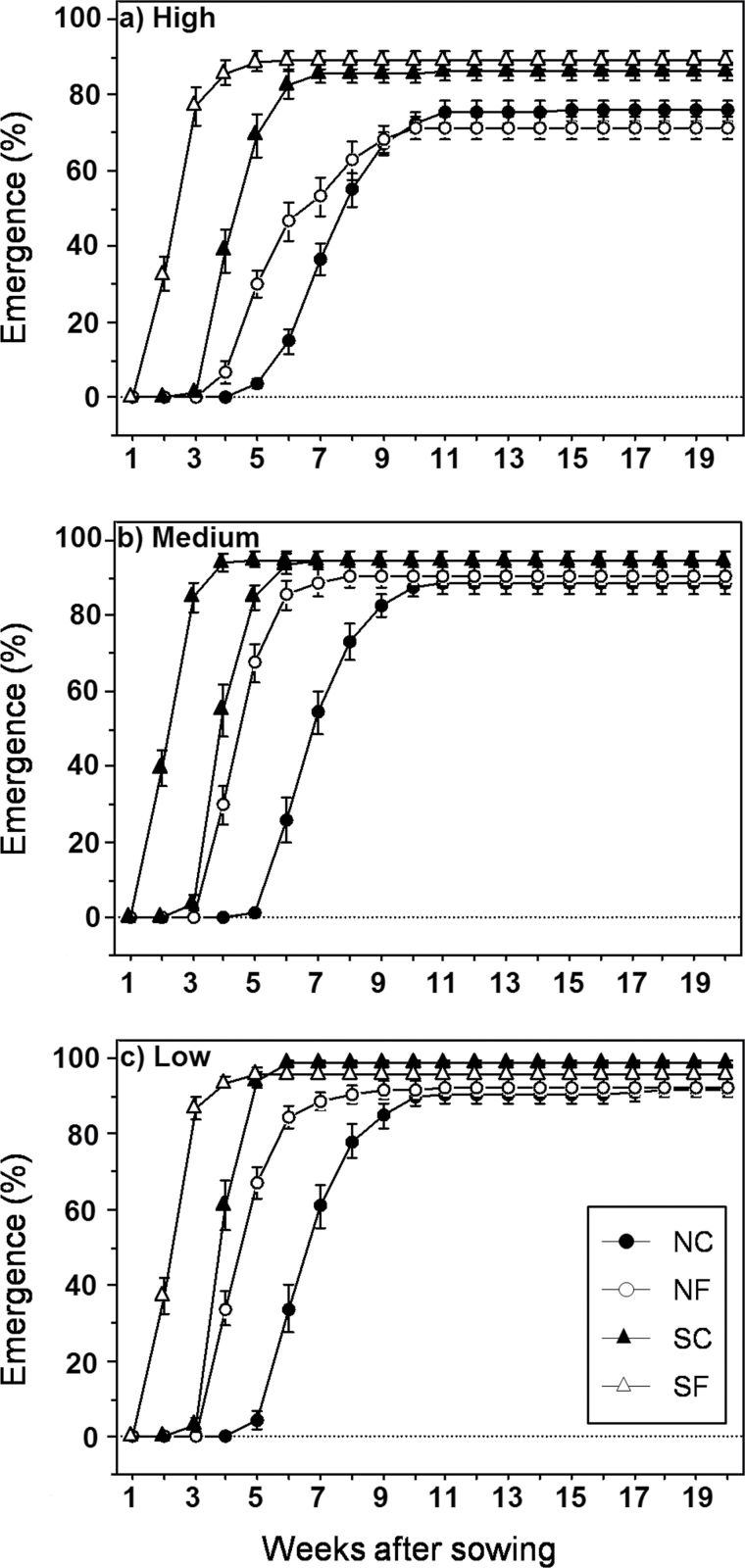
Cumulative emergence over time across precipitation levels: high (a), medium (b), low (c), and temperatures (NC, NF, SC, and SF). For simplicity, both provenances are pooled. Results are given as means ±SE.

A total of 1931 seedlings emerged, starting 8 d after sowing. Both emergence time and proportion were affected by the three main experimental factors (Table 3). Temperature was the main factor controlling emergence time. At both limits, increased temperature reduced the mean emergence time by about 14 d with northern temperatures and by 12 d with southern temperatures (Fig. 2). In addition, southern temperatures enhanced emergence proportion compared with northern ones (overall 93.5±1.3 vs 85.3±0.9%; *P*<0.0001), although the temperature increase expected for the coming decades within each range limit had no effect, as NF and SF treatments were comparable to NC and SC treatments, respectively. Seeds from southern populations emerged faster (32.2±1.1 vs 36.0±1.3 d) and in a higher proportion (91.8±1.1 vs 87.0±1.3%) than those from the northern limit (Table 3). Precipitation also affected emergence time and proportion. Overall, higher precipitation increased the time to emerge and reduced the emergence proportion, whereas no differences appeared between medium and low treatments (Table 3, Fig. 2). In addition, temperature had significant interactions with provenance and precipitation for emergence time and with precipitation for emergence rate (Table 3).

**Table 3.** Summary of statistics (χ^2^ or F and P values)Emergence and survival rates were analysed by a logistic model (χ^2^ values), and differences in emergence time, total biomass, and root:shoot ratio by the same model but with normal error distribution and identity as a link function (F values). DF, degrees of freedom.

	χ^2^/F	*P*	DF
**Time to emergence**			
Temperature (T)	822.3	<0.0001	3
Provenance (Pr)	62.4	<0.0001	1
Precipitation (Pt)	29.9	<0.0001	2
T×Pr	4.3	0.006	3
T×Pt	6.1	<0.0001	6
Pr×Pt	0.02	0.98	2
T×Pr×Pt	0.87	0.51	6
**Emergence rate**			
T	36.3	<0.0001	3
Pr	10.1	0.0015	1
Pt	67.6	<0.0001	2
T×Pr	7.4	0.06	3
T×Pt	7.9	0.24	6
Pr×Pt	3.4	0.19	2
T×Pr×Pt	12.9	0.045	6
**Survival**			
T	152.9	<0.0001	3
Pr	23.7	<0.0001	1
Pt	28.5	<0.0001	2
T×Pr	10.2	0.017	3
T×Pt	127.8	<0.0001	6
Pr×Pt	0.04	0.97	2
T×Pr×Pt	3.9	0.68	6
**Total biomass**			
T	275.2	<0.0001	3
Pr	39.4	<0.0001	1
Pt	51.5	<0.0001	2
T×Pr	2.0	0.11	3
T×Pt	61.6	<0.0001	6
Pr×Pt	0.1	0.87	2
T×Pr×Pt	0.7	0.66	6
**Root:shoot ratio**			
T	120.8	<0.0001	3
Pr	254.2	<0.0001	1
Pt	5.93	0.003	2
T×Pr	0.95	0.42	3
T×Pt	4.25	0.0005	6
Pr×Pt	0.38	0.68	2
T×Pr×Pt	1.93	0.08	6

### Seedling survival

**Fig. 3.**
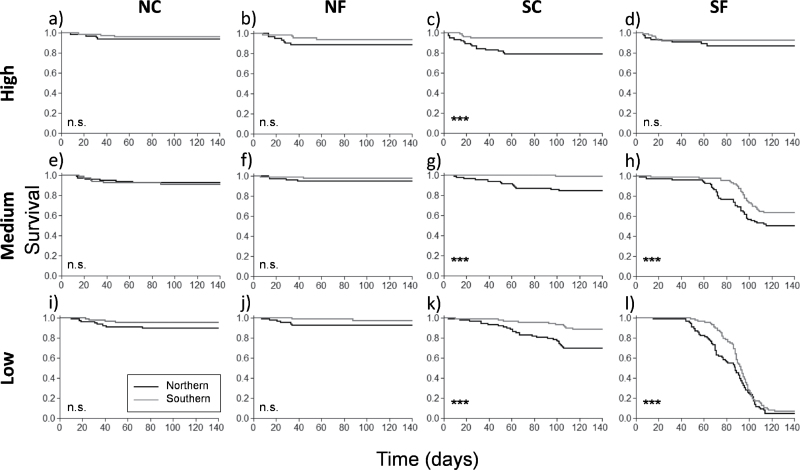
Survival probabilities of Scots pine seedlings from northern (black line) and southern (grey line) provenances growing under the different combinations of temperature (columns) and precipitation (rows) levels during experiment development. ***, statistically significant differences between provenances at *P*<0.0001; n.s., non-significant differences, from a log-rank test.

From the emerged seedlings, 1551 survived at the end of the experiment. Although the overall survival was high (80%), it was strongly affected by the three main factors (Table 3). Temperature was the factor with the strongerst influence on seedling survival, but the consequences of the increased temperatures were different if applied to the northern or southern conditions: whereas a temperature increase slightly enhanced the survival of Scots pine seedlings growing under the northern conditions (Table S1, available at *JXB* online), it strongly reduced the survival rate when applied to the southern conditions (Fig. 3). Seedlings from the southern provenance also had higher survival rates than northern ones (84.6±2.7 vs 77.4±2.8&), under both northern and southern conditions. Precipitation amount had an overall positive effect on survival, rising from the low level (68.1±4.6%) to medium (84.2±2.9%) and high (90.7±1.5%). However, differences among precipitation levels were stronger as temperature increased (Fig. 3), as denoted by the significant interaction between temperature and precipitation (Table 3).

### Biomass and allocation pattern

**Fig. 4.**
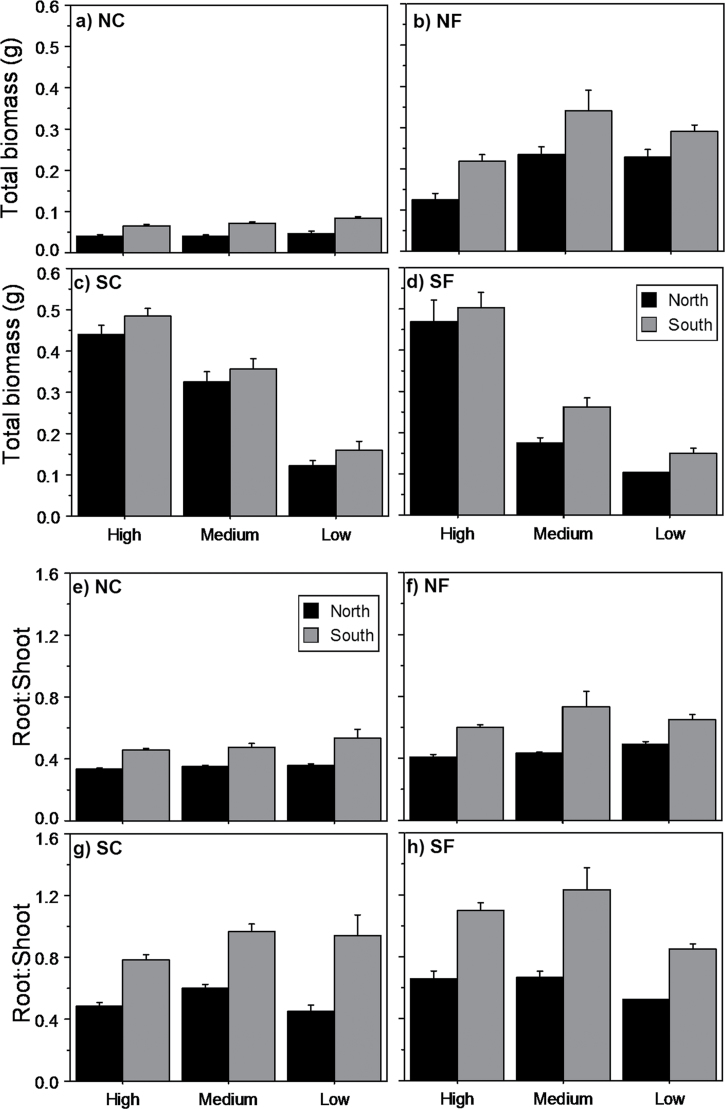
Total biomass (a–d) and root:shoot ratios (e–h) of seedlings from northern (black bars) and southern (grey bars) provenances across the different levels of precipitation (high, medium, and low) and temperature (NC, NF, SC, and SF). Results are given as means ±SE.

As in the case of emergence time and survival, final biomass at the end of the experiment was controlled mainly by temperature (Table 3). Under the conditions of both range limits, increased temperatures enhanced total biomass, this pattern being especially clear for the northern climate (Fig. 4a–d). In addition, increased temperatures expected at both range limits induced a higher allocation of biomass to roots. Precipitation also affected biomass, but the effect was different under northern and southern conditions. Whereas under southern temperatures (both current and future) higher precipitation led to higher biomass, under northern temperatures, the high precipitation level had no effect on the final weight. Finally, provenance affected root length (168.9±4.1mm vs 190.2±3.6mm for northern and southern provenances, respectively; *P*<0.0001), total biomass and, especially, root:shoot ratio. Seeds from the southernmost distribution of the species produced heavier seedlings than those from the northern limit. Moreover, provenance was the main factor affecting biomass allocation, with seedlings from a southern origin investing more in belowground parts than northern ones (Fig. 4e–h). This pattern among provenances was maintained across climate conditions, as there were no significant interactions between provenance and temperature or precipitation for final biomass or root:shoot ratio (Table 3).

**Fig. 5.**
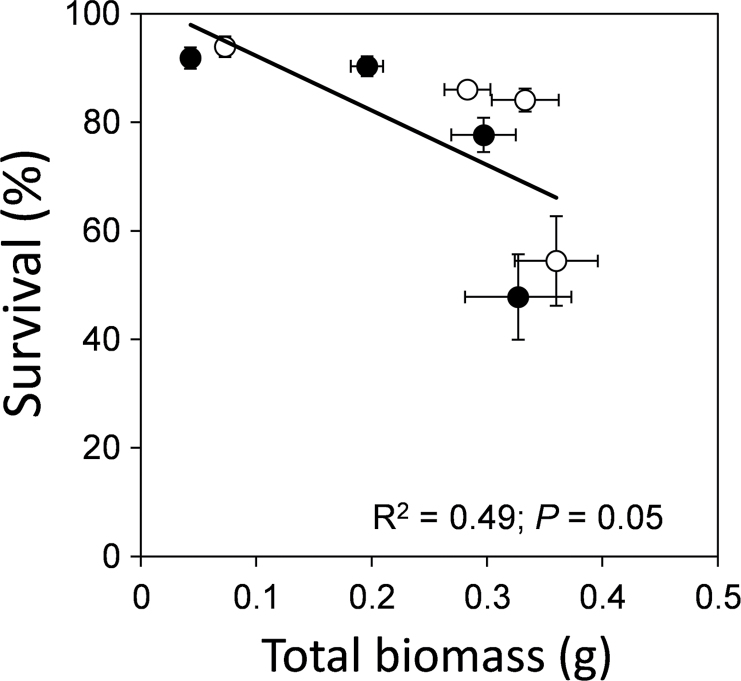
Growth (expressed as total biomass at the end of the experiment) and survival relationships of seedlings from the two provenances (black circles, northern; white circles, southern) among the different temperature scenarios. *R* ^2^ and *P* values are indicated on the figure. Error bars represents ±SE.

Absolute growth, expressed as total biomass at the end of the experiment, and survival were negatively related (*R* ^2^=0.49; *P*=0.05) among the different temperature levels (Fig. 5). Higher temperature enhanced growth, but reduced the survival probability, resulting in a phenotypic trade-off between these two processes.

## Discussion

The results obtained in this study support our hypothesis that recruitment will be significantly reduced at the southernmost range of the species, in terms of both biomass gain and survival, whereas it will be enhanced at the northern limit. In addition, we determined that the southern seed sources are better adapted to survive under drier conditions, and that the future climate will impose a trade-off between seedling growth and survival probabilities.

### Recruitment dynamics at range limits

Latitudinal range limits of Scots pine distribution are highly susceptible to impacts of altered climatic conditions predicted over the coming decades. At both limits, rising temperatures reduced the time to emergence, which may have several positive consequences for seedling success. An early emergence allows seedlings a larger time span for development before the onset of adverse conditions (either cold at the northern range or drought at the southern range). This implies several advantages, such as the vigorous growth of the root system, better interception of light, and/or better intra- and inter-specific competitive performance (Ross and Harper, 1972; Miller, 1987; Baskin and Baskin, 1998; Seiwa, 2000; Verdú and Traveset, 2005). Thus, we can expect that the future climate will enhance the early stages of Scots pine regeneration at both range limits.

At the northern limit of the species, however, the expected temperature increase had little effect on emergence proportion and growth, whereas survival was only slightly enhanced by increased precipitation. This weak effect of precipitation in the different phases of recruitment is an expected result, as water is not a limiting factor during growing season at the northern limit (Moberg *et al.*, 2005). More importantly, however, increased temperature strongly influenced seedling growth. Higher biomass gain at the end of the growing season confers a higher probability that seedlings will survive the winter frost and, in successive years, increases the probability of escape from browsers and reduces the time to reproduction, ultimately accelerating the regeneration process (Holtmeier, 2005; Mathisen and Hofgaard, 2011). As Scots pine growth is restricted mainly by cold at the northernmost limit (Rickebusch *et al.*, 2007; Salminen and Jalkanen, 2007), these results provide evidence that the predicted warming is likely to diminish this limitation, which, together with the high emergence and survival rates, will result in significantly enhanced regeneration. This pattern is in concordance with the recruitment increase already recorded for this species close to the treeline in the Swedish Scandes and in northern Finland during the last decades as a response to a progressive rise in temperatures (Stöcklin and Körner, 1999; Kullman, 2001, 2002; Juntunen *et al.*, 2002; Holtmeier, 2005; Juntunen and Neuvonen, 2006; Holtmeier and Broll, 2011), thereby leading to future range changes (Matías and Jump, 2012).

In contrast, predicted changes in temperature at the southernmost range limit will have an overall negative impact on Scots pine regeneration. Although the increased temperature slightly boosted the biomass gain, it almost halved survival probability. In addition, the expected reductions in precipitation further decreased growth rate and reduced survival probability to values close to zero when the increased temperature and reduced precipitation treatments were combined. Consequently, changes in temperature and precipitation act synergistically in the same direction by a reduction in the total water input and by an increase in evapotranspiration loss, as denoted by the significant interaction between these two factors. Water availability during the summer has been proven to be the key factor driving Scots pine regeneration at the southernmost limit of its distribution (Castro *et al.*, 2005; Matías *et al.*, 2011*b*), as supported by our results. Thus, as long as current trends in climate change are maintained during the coming decades, recruitment in this species could be seriously constrained by a reduction in both seedling growth and survival probability, with recruitment being completely absent during the driest years (Mendoza *et al.*, 2009; Matías *et al.*, 2012*a*). This hampered recruitment, together with the high mortality rates that are being recorded for this species at the southern range (Martínez-Vilalta and Piñol, 2002; Galiano *et al.*, 2010; Sanchez-Salguero *et al.*, 2012), is likely to result in rapid population decline and in a shift in dominance to other drought-tolerant species such as Mediterranean *Quercus* spp. (Galiano *et al.*, 2010; Matías *et al.*, 2012*a*).

### Local adaptations to climate

Seedlings from the southern range of the species emerged faster than the northern ones. The benefits of an early emergence (outlined above) are that it allows southern Scots pine seedlings to produce a well-developed root system before the onset of summer drought, which enhances their survival probabilities (Castro, 2006). In contrast, a delayed emergence may be positive at the northern range, as it reduces the possibility of premature germination and late frost damage (Cavieres and Arroyo, 2000; Milbau *et al.*, 2009). Seeds produced at the northern and southern ranges also differ in weight (Table 1). Higher seed mass, as those from the southern provenance had, has been positively related to emergence rate and early growth for this species (Castro, 1999; Debain *et al.*, 2003). Larger seeds retain a greater proportion of their seed reserves after germination, which can be mobilized for growth, producing larger seedlings able to overtop neighbouring seedlings and capture more light and to explore deeper soil, ultimately bolstering establishment probabilities when environmental conditions are adverse (Foster, 1986; Poorter and Hayashida-Oliver, 2000; Green and Juniper, 2004). However, the most significant difference among range limits is the differential biomass allocation pattern. Southern seedlings invest a higher proportion of their biomass in the root system than northern ones, irrespective of the temperature range in which they are grown. A longer and more developed root system is a key trait to cope with summer drought (Collins and Bras, 2007; Markesteijn and Poorter, 2009). It allows both the uptake of water from deeper and moister soil profiles and higher reserve storage for the dry period (Lloret *et al.*, 1999; Paula and Ojeda, 2009), translating ultimately into a higher survival probability at the southernmost range. Scots pine populations have been generally described to perform better in their own environment than other genetic sources in general (Rehfeldt *et al.*, 2002; Reich and Oleksyn, 2008), and Mediterranean populations in particular do better than continental ones (Richter *et al.*, 2012), as also confirmed by our results. Although it should be noted regarding local adaptation that only high temperature and precipitation effects were investigated here, other factors such as the possible greater resistance of northern seedlings to frost and lower temperatures deserve additional investigation.

### Trade-off implications

Projected changes in environmental conditions will have contrasting consequences for Scots pine regeneration. Higher temperatures have an overall clear positive effect by the reduction in emergence time and by the increase in growth rate. However, the expected reduction in precipitation at the southern range, together with the higher evapotranspiration as a consequence of increased temperature, implies a strong reduction of survival probabilities. These opposing effects result in a trade-off between growth and survival, where the best scenario for one of the processes is strongly negative for the other. Our growth calculations are based only on survivors and may overestimate true population growth. However, this type of relationship among seedling growth and survival is common for many species among resource gradients (Dalling and Hubbell, 2002; Engelbrecht and Kursar, 2003, Seiwa, 2007), and its final balance is often determined by the biomass allocation pattern (Smith and Huston, 1989). As we have seen, seedlings from the southern provenance invest more biomass in rooting than northern ones, which, together with the above-described advantages of an earlier emergence, might confer higher probabilities to survive under an increased drought. However, overall at the population level, deleterious effects on survival are much more important than any positive effect on growth, as they may completely hamper recruitment at the southernmost limit for this species (Castro *et al.*, 2005; Mendoza *et al.*, 2009; Matías *et al.*, 2012*a*; Richter *et al.*, 2012).

### Conclusions

According with our results, latitudinal range limits of Scots pine distribution are highly susceptible to alteration by the changes in climate predicted for the coming decades. At the northern limit, growth limitation and cold damage might be alleviated by climate warming, resulting in enhanced growth and survival. However, at the southern range edge, warming is likely to increase heat stress and exacerbate the water deficit already elevated due to the reduced precipitation and increased evapotranspiration, leading to significantly decreased growth and survival. Thus, these changes support the predicted general retreat of this species’ rear edge and its northward and upward displacement. However, it is important to note that the detected early emergence in response to warming may modulate this general pattern by enhancing survival at the southern limit and by increasing the probability of frost damage at the northern range. Consequently, the model of a simple temperature-driven shift towards higher altitudes and latitudes is likely to be an oversimplification for this species. This difference in climate sensitivity of particular stages of the establishment process, together with the high inter-annual variability in climate and the presence of sporadic extreme climatic events, adds significant uncertainty to the forecasting of range shifts. Furthermore, although it has been reported that Scots pine seedlings have a very low drought resistance when compared with other cohabiting Mediterranean species (Matías *et al.*, 2012*b*) and that southernmost populations are imperilled under the expected climate (Benito Garzón *et al.*, 2008; Matías *et al.*, 2012*a*), local adaptations may play an important role, allowing the persistence of the species in relict areas where microclimatic and topographical conditions are favourable for survival (Hampe and Jump, 2011). Finally, the better performance of seedlings from southern populations outside their native conditions suggests that they might be a valuable genetic resource for enhancing natural resilience to climate change in highly susceptible areas (see McLachlan *et al.*, 2007, for a debate), and highlights the resource-conservation value of these southernmost relict populations (Jump *et al.*, 2009*b*; Hampe and Jump, 2011).

## Supplementary data

Supplementary data are available at *JXB* online.

Supplementary Table S1. Mean values (±SE) of the studied variables across the different temperature and precipitation scenarios.

Supplementary Data

## Abbreviations:

ANOVA: analysis of variance
NC: north current: current main temperature during growing season at the northern limit of the species
NF: north future: predicted temperature by 5 °C at the northern limit for the end of the present century
SC: south current: current main temperature during growing season at the southern limit of the species
SE: standard error
SF: south future: predicted temperature by 5 °C at the southern limit for the end of the present century.
