# Supplementary material for: Impacts of predicted climate change on recruitment at the geographical limits of Scots pine
Source: J Exp Bot. 2013 Nov 12;65(1):299–310. doi: 10.1093/jxb/ert376 (PMC3883299; doi:10.1093/jxb/ert376)
Supplement: Supplementary Data [file supp_65_1_299__index.html]

Impacts of predicted climate change on recruitment at the geographical limits of Scots pine — Impacts of predicted climate change on recruitment at the geographical limits of Scots pine — Supplementary Data 

# Impacts of predicted climate change on recruitment at the geographical limits of Scots pine

## Supplementary Data

Data files

**Files in this Data Supplement:**

- Supplementary Data - Supplementary Data
