# Supplementary material for: Impacts of predicted climate change on recruitment at the geographical limits of Scots pine
Source: J Exp Bot. 2013 Nov 12;65(1):299–310. doi: 10.1093/jxb/ert376 (PMC3883299; doi:10.1093/jxb/ert376)
Supplement: Supplementary Data [file supp_ert376_jexbot102830_file001.pdf]

Impacts of predicted climate change on recruitment at the geographical limits of Scots pine

Luis Matias and Alistair S Jump

Supplementary Data S1: Mean values (±SE) of the studied variables across the different temperature and precipitation scenarios (NC: current temperature at the northern limit of the species; NF: expected future temperature at the northern limit; SC: current temperature at the southern limit; SF: expected future temperature at the southern limit).

	Emergence time (days)	Emergence rate (%)	Survival (%)	Total biomass (g)	Root:shoot
Northern provenance					
NC	53.4 ± 0.9	82.6 ± 2.2	91.8 ± 2.0	0.043 ± 0.002	0.35 ± 0.01
NF	39.7 ± 1.3	83.3 ± 3.3	92.3 ± 1.8	0.196 ± 0.014	0.44 ± 0.01
SC	32.5 ± 0.6	92.6 ± 2.0	77.7 ± 3.2	0.297 ± 0.028	0.51 ± 0.02
SF	18.2 ± 0.5	90.0 ± 2.1	47.8 ± 7.9	0.327 ± 0.046	0.66 ± 0.03
High	38.5 ± 2.4	76.9 ± 2.2	87.1 ± 2.4	0.269 ± 0.035	0.47 ± 0.02
Medium	34.6 ± 2.2	90.0 ± 2.0	81.0 ± 4.2	0.195 ± 0.020	0.50 ± 0.02
Low	34.8 ± 2.1	94.2 ± 1.4	64.1 ± 6.3	0.132 ± 0.016	0.44 ± 0.02
Southern provenance					
NC	48.4 ± 0.9	88.9 ± 2.5	93.9 ± 1.7	0.073 ± 0.003	0.49 ± 0.02
NF	34.6 ± 0.9	86.7 ± 2.5	96.0 ± 1.4	0.283 ± 0.020	0.66 ± 0.03
SC	28.3 ± 0.5	94.4 ± 1.5	94.1 ± 2.1	0.333 ± 0.029	0.90 ± 0.05
SF	17.3 ± 0.3	97.0 ± 1.0	54.4 ± 8.2	0.360 ± 0.036	1.14 ± 0.07
High	34.8 ± 2.2	85.0 ± 2.1	94.3 ± 1.4	0.318 ± 0.033	0.74 ± 0.04
Medium	30.9 ± 1.9	94.7 ± 1.6	87.4 ± 3.9	0.258 ± 0.024	0.85 ± 0.06
Low	30.7 ± 1.8	95.6 ± 1.2	72.1 ± 6.6	0.175 ± 0.018	0.72 ± 0.06
